# Berberine Modulates LPA Function to Inhibit the Proliferation and Inflammation of FLS-RA via p38/ERK MAPK Pathway Mediated by LPA_1_

**DOI:** 10.1155/2019/2580207

**Published:** 2019-11-03

**Authors:** Hui Wang, Shenghao Tu, Sisi Yang, Pan Shen, Yao Huang, Xin Ba, Weiji Lin, Ying Huang, Yu Wang, Kai Qin, Zhe Chen

**Affiliations:** ^1^Institute of Integrated Traditional Chinese and Western Medicine, Tongji Hospital, Tongji Medical College, Huazhong University of Science and Technology, Wuhan, Hubei 430030, China; ^2^Division of Geriatrics, Tongji Hospital, Tongji Medical College, Huazhong University of Science and Technology, Wuhan, Hubei 430030, China

## Abstract

**Objective:**

This study aimed to investigate whether berberine exerted anti-inflammatory and antiproliferative effects on the fibroblast-like synoviocytes of rheumatoid arthritis (FLS-RA) through regulating the lysophosphatidic acid (LPA) function.

**Methods:**

Firstly, the expression levels of LPA and lysophosphatidic acid receptor 1 (LPA_1_) in RA patients, osteoarthritis (OA) patients, and healthy controls were detected. Moreover, molecular docking was employed to characterize the binding sites of berberine in the predicted protein targets. Later, FLS-RA were stimulated using berberine, LPA, and the specific inhibitor (Ki16425) of LPA_1,_ thereafter, the effects on the proliferation, apoptosis, the release of inflammatory mediators of FLS-RA, and the MAPK pathway were observed.

**Results:**

Compared with healthy controls (*n* = 25), the plasma LPA level (*n* = 28) and synovial fluid (*n* = 10) were markedly higher in RA patients. LPA_1_ was highly expressed in RA patients (*n* = 4) relative to that in OA patients (*n* = 4). Berberine remarkably inhibited the proliferation and the excessive production of IL-6 and TNF-*α* in FLS-RA, whereas suppressing the expression of K-ras, c-Raf, and p-38/ERK-phosphorylation. In addition, berberine inhibited the LPA-induced p-38/ERK-phosphorylation through binding to LPA_1_.

**Conclusions:**

LPA plays a certain role in promoting the proliferation and inflammation of FLS-RA. Berberine potentially modulates LPA function to suppress the proliferation and inflammation of FLS-RA through blocking the p38/ERK MAPK pathway mediated by LPA_1_. These findings suggest that, berberine possesses potential lipid-regulating, antiarthritis, and synovial hyperplasia inhibition activities against RA, which may provide a promising therapeutic target for the clinical drug development for RA patients with dyslipidemia and high CVD risk.

## 1. Introduction

Rheumatoid arthritis (RA) is a destructive arthropathy characterized by chronic synovial inflammation and hyperplasia, which results in multijoint failure, ultimately affecting approximately 1% of the total population [1, 2]. Fibroblast-like synoviocyte (FLS), a synovium component, has been regarded as the main effector cell during the process of disease development, which releases metal matrix protease and formats the inflammatory cascade. Epidemiological studies have suggested that cardiovascular disease (CVD) is one of the leading causes of death among RA patients, and the risk of CVD mortality is increased by 50–60% in RA patients compared with that in general population [3–6]. In addition, evidence supports that high inflammatory burden and dyslipidemia are associated with an increased CVD risk in RA [7]. Lipid metabolism disorders, including changes in lipid level and compositional distribution, have been discovered throughout the course of RA [8]. Traditionally, inflammation alters the lipid levels, but the effect of lipids on arthritis remains incompletely known so far.

Lysophosphatidic acid (LPA), a small ubiquitous lipid that shows direct correlation with atherosclerosis, has been detected visibly to be increased in RA, which has also been shown to be a potent lipid-signaling medium [9]. In inflammatory state, LPA is mainly produced through the decomposition of lysophosphatidylcholine by lysophospholipase D (also known as autotaxin, ATX) in the activated platelets. In addition, LPA is the active compound in the mildly oxidized low-density lipoprotein (LDL) and the minimally modified LDL. The LPA signaling is suggested to be involved in the inflammatory response of FLS. Additionally, it is also indicated to evoke the growth factor-like responses in the proliferation and apoptosis of FLS-RA, which is achieved through activating the lysophosphatidic acid receptor and the mitogen-activated protein kinase (MAPK) signaling. Additionally, the interaction between LPA and inflammation may accelerate RA progression and increase the CVD risk in RA [10].

Berberine is a quaternary ammonium alkaloid existing in several natural herbal medicines. According to the modern pharmacological research, berberine has antibiotic and lipid-regulating effects [11–13]. Our previous results also indicate that berberine remarkably alleviates arthritis in rats with bovine type II collagen-induced arthritis (CIA) [14]. However, it remains unclear whether such effect of berberine is due to the direct action on FLS or is achieved indirectly by reducing inflammatory cytokines, as well as whether berberine regulates lipid and inhibits inflammation. It is conjectured that berberine may downregulate the MAPK pathway through regulating the function of LPA signaling and exert the anti-inflammatory and preventive effects on arteriosclerosis, thus improving RA and reducing the risk of RA-CVD.

## 2. Materials and Methods

### 2.1. Patients

Human synovial tissues, peripheral blood, and synovial fluid were obtained during the joint replacement surgery; meanwhile, sections were obtained from outpatients at Tongji Hospital (Wuhan, China). This study followed the guidelines of the Declaration of Helsinki and Tokyo for humans. All RA participants were diagnosed in strict accordance with the 1987 criteria of American College of Rheumatology, and they had signed an informed consent approved by the hospital ethics committee (no. TJ-IRB20181117). Routine blood tests were carried out using the XE 5000 instrument (Sysmes).

### 2.2. Cell Isolation and Culture

Primary FLS-RA were isolated from the synovial tissues of RA (*n* = 4) donors. Afterwards, the fresh synovial tissues were washed with phosphate-buffered solution (PBS) three times and cut into small pieces < 1 mm^3^. Then, the synovial biopsies were treated with 2 mg/ml of type 2 collagenase for about 2 h, and the mixture was filtered with the 300-mesh cell sieve. Later, DMEM (Hyclone, SH30022.01) containing 10% fetal bovine serum (FBS, Gibco, 42Q0082K) was added into the flasks, placed into the 5% CO_2_ thermostatic incubator at 37°C, and splitted at a ratio of 1 : 3. Then, the 3rd–6th generations of cells were used in experiments. All FLS-RA were starved for 6 h with serum-free medium prior to subsequent experiments.

### 2.3. Histopathology and Immunohistochemistry (IHC)

First of all, both RA (*n* = 4) and OA (*n* = 4) synovial tissues were sectioned and stained using hematoxylin and eosin (H&E), followed by formalin fixation and paraffin embedding. Later, the RA and OA synovial tissue sections were deparaffinized, treated with citrate buffer at 80°C for 30 min, and washed with H_2_O. Afterwards, endogenous peroxidase was blocked with 1% H_2_O_2_ for 10 min. Then, the slides were blocked with 2% goat serum for 1 h and incubated at 4°C with a rabbit polyclonal antibody (Ab) against LPA_1_ (Abcam, ab23698) overnight. After washing with PBS twice, all slides were incubated with the respective biotinylated secondary Abs for 30 min. Later, the signals were amplified under horseradish peroxidase (HRP) conjugation, and finally, the slides were developed using the chromogenic substrate for peroxidase.

### 2.4. Enzyme-Linked Immunosorbent Assay (ELISA) and Platelet Detection

The LPA contents in RA plasma samples (*n* = 28), healthy controls (HC, *n* = 25), and synovial fluid of RA patients (SF-RA, *n* = 10) were detected and compared according to the manufacturer instructions (Bio-Swamp, HMI12216).

### 2.5. MTT Assay

FLS-RA (6 *∗* 10^5^ cells/ml) were seeded into the 96-well plates, and all cells were pretreated with 10 ng/ml IL-1 for 8 h. When screening the suitable berberine (Shifeng, C20H18NO4) content, cells were grown in the mediums supplemented with various berberine doses (0, 6.25, 12.5, 25, 50, and 100 *μ*M) for 12 h, 24 h, and 48 h, respectively. When screening the appropriate LPA (Sigma, L7260) content, cells were planted in the medium containing various LPA doses (0, 5, and 10 *μ*M) for 24 h, respectively. When selecting the suitable Ki16425 (Cayman, 10012659) content, cells were plated with various Ki16425 doses (0, 10, and 20 *μ*M) for 24 h, respectively. Afterwards, the cells were incubated with berberine (12.5 and 25 *μ*M) for 1 h and then treated with 10 *μ*M LPA or Ki16425 for 24 h, so as to investigate the mechanism of berberine in inhibiting cell proliferation. Subsequently, the wells were incubated with 1 mg/ml MTT (3-(4,5-dimethylthiazol-2-yl)-2,5-diphenyltetrazolium bromide; Sigma, m2128) at 37°C for 4 h, and then 100 *μ*L dimethyl sulfoxide was added into each well. After the purple formazan crystals were completely dissolved, the optical density (OD) was measured at the wavelength of 490 nm.

### 2.6. Apoptosis and Cell Cycle Assays

Flow cytometry (BD) was employed for the apoptosis and cell cycle assays. To be specific, FLS-RA (1.5 *∗* 10^5^ cells/well) were seeded into the 6-well plates and treated with berberine (12.5 and 25 *μ*M) for 24 h. Then, cells were digested with EDTA-free trypsin, and a portion of cells were stained with 7-AAD and Annexin V (BD, 559763) for 30 min in dark at room temperature for apoptosis assay; meanwhile, the other portion of cells were stained with PI for cell cycle assay in accordance with the manufacturer instructions. The FlowJo software was used for analyses.

### 2.7. Molecular Docking

To characterize the binding sites of berberine in the predicted protein targets (LPA_1_, PBD ID: 4z34), molecular docking was carried out using the Autodock, Chimera, Pymol, and Avogadro software. Then, the 3D structures of LPA_1_ and berberine were applied, and the binding site of the cocrystallized ligand was taken as the starting structure to predict the binding affinity of berberine to the protein.

### 2.8. Reverse Transcription Polymerase Chain Reaction (RT-PCR)

FLS-RA (1.5 *∗* 10^5^ cells/well) were seeded into the 6-well plates, and then treated with berberine (12.5 and 25 *μ*M) for 24 h, respectively. In the experiment to verify the binding of berberine to LPA_1_, cells were incubated with berberine (12.5 and 25 *μ*M) for 1 h, respectively, followed by 24 h treatment with 10 *μ*M LPA or Ki16425. Afterwards, the TRIzol reagent (Takara, 108-95-2) was added in each group to isolate the total RNA. Then, the reverse transcription reaction was conducted using the specific kit according to the manufacturer protocols (Takara, RR820A). The specific primers used for individual target cDNA are shown below, among which, *β*-Actin was used as an internal control to normalize the relative expression of TNF-*α*, IL-6, K-ras, and autotaxin (ATX, ENPP2).

(H-ATX-ENPP2-F: CGTGTTTCTCCGAGTTTCAGTC, H-ATX-ENPP2-R: CTCCATTTCTTTCCGAAGCATA; H-KRAS-F: TGTCCCCACGGTCATCCA; H-KRAS-R: CACCACCCCAAAATCTCAACT; H-IL6-F: CAATAACCACCCCTGACCCA; H-IL6-R: CATGCTACATTTGCCGAAGAG; H-TNF-F: CCCTCCTTCAGACACCCTCA; and H-TNF-R: GTGGTTGCCAGCACTTCACT).

### 2.9. Western Blotting

FLS-RA (8 *∗* 10^5^ cells/well) were seeded into a dish that was 10 cm in diameter. Then, cells were treated with berberine (12.5 and 25 *μ*M) for 24 h to obtain c-Raf and phosphorylated protein. In the experiment to verify the binding of berberine to LPA_1_, cells were incubated with berberine (12.5 and 25 *μ*M) for 1 h prior to 24 h treatment with 10 *μ*M LPA. Afterwards, protein was extracted using RIPA (200 *μ*L for each dish) and cocktail, and equivalent amounts of the protein were subjected to electrophoresis on 12% SDS acrylamide. After electrophoresis, the proteins were transferred onto the NC membranes and blocked with 5% skim milk for 2 h at room temperature to get rid of the nonspecific binding. Blots were incubated with the primary antibody against c-Raf (Cell Signaling Technology, 9422S), phospho-p38 (P-p38, Cell Signaling Technology, 4511S), phospho-ERK1/2 (P-ERK, Cell Signaling Technology, 4370S), phospho-JNK (P-JNK, Cell Signaling Technology, 9255S), tubulin (Cell Signaling Technology, 5568T), and GAPDH (Cell Signaling Technology, 5274T) at 4°C overnight. After washing with TBST, the blots were further incubated with anti-rabbit/mouse IgG for 1 h at room temperature. Finally, the blots were developed with the Western detection reagents (Odyssey).

### 2.10. Statistical Analysis

GraphPad prism 5.0 was applied for all statistical analyses. Data were expressed as mean ± S.D. The one-way analysis of variance (ANOVA) was adopted for multiple comparisons, whereas linear regression was utilized for correlation analysis. A difference of ^*∗*^*P* < 0.05 was considered as statistically significant.

## 3. Results

### 3.1. The Levels of Plasma LPA and Synovial Lysophosphatidic Acid Receptor 1 (LPA_1_) in RA Patients Were Markedly Higher than Those in Controls

**Figure 1:**
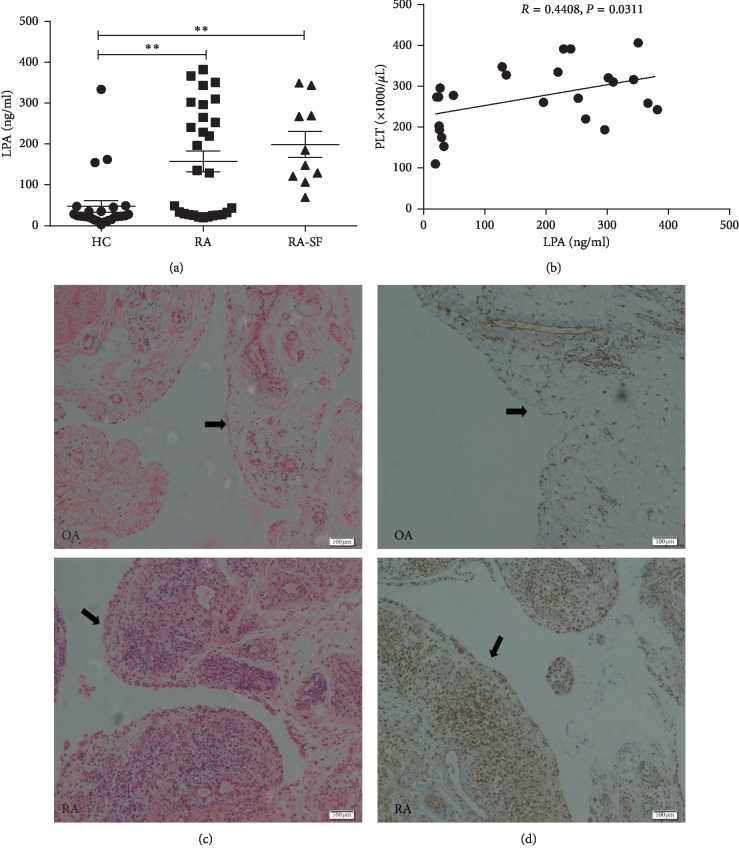
The levels of plasma LPA and synovial LPA_1_ in RA patients are evidently higher than those in controls. (a) The plasma LPA levels measured by ELISA in RA (*n* = 28) are dramatically higher than those in healthy controls (*n* = 25), but there is no statistical difference between RA and RA-SF (*n* = 10). (b) The plasma LPA level (*n* = 23) shows positive correlation with the platelet level. (c) Visible synovial thickening is observed in RA patients compared with OA patients (*n* = 4) in the light of H&E staining (×100). (d) The IHC staining acquired from the knee joints of RA and OA patients is shown (×100), LPA_1_ is expressed in the synovial tissues of both RA and OA patients, and higher levels are observed in RA cases (*n* = 4). ^*∗∗*^*P* < 0.01. LPA, lysophosphatidic acid; RA, rheumatoid arthritis; HC, healthy control; RA-SF, synovial fluid of RA; OA, osteoarthritis; and PLT, platelet.

The plasma LPA levels in RA patients (*n* = 28), HCs (*n* = 25), and synovial fluid from RA patients (RA-SF, *n* = 10) were analyzed by ELISA. As shown in Figure 1(a), the plasma LPA levels in RA were dramatically higher than those in health controls (*P* < 0.01), but there was no statistical difference between RA and RA-SF. In addition, the plasma LPA level was positively correlated with the platelet level (Figure 1(b), *R* = 0.4408, *P*=0.0311). Meanwhile, compared with OA patients (*n* = 4), visible synovium thickening was observed in RA patients (*n* = 4, Figure 1(c)) based on H&E staining. Moreover, LPA_1_ was expressed in the synovium of both RA and OA patients, but higher levels were detected in RA cases (Figure 1(d)).

### 3.2. Berberine Inhibited the Growth and Secretion of Proinflammatory Factors and Promoted the Apoptosis of FLS-RA

**Figure 2:**
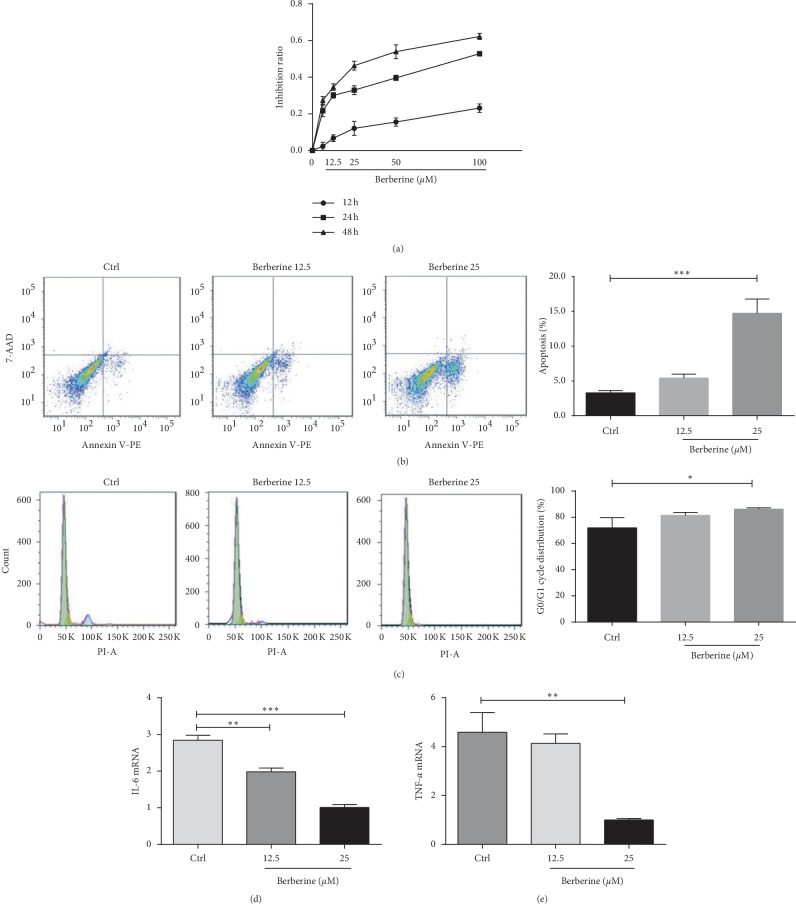
Berberine suppresses the growth and secretion of proinflammatory factors and promotes the apoptosis of FLS-RA. (a) Berberine inhibits FLS-RA proliferation in a time- and dose-dependent manner, as detected by MTT assay. (b) Berberine treatment at 12.5 and 25 *μ*M for 24 h promotes the early apoptosis of FLS-RA, as detected by flow cytometry. (c) Berberine treatment at 12.5 and 25 *μ*M for 24 h prevents cells from dividing to render cell cycle arrest at the G0/G1 phase, as measured by flow cell cycle detection. (d) Berberine treatment at 12.5 and 25 *μ*M for 24 h evidently inhibits the mRNA expression of IL-6 in a dose-dependent manner, as detected by RT-PCR. ^*∗*^*P* < 0.05, ^*∗∗*^*P* < 0.01, and ^*∗∗∗*^*P* < 0.001. Ctrl, control group. (e) The berberine dose of 25 *μ*M obviously inhibited the mRNA expression of TNF-α, as detected by RT-PCR.

The fusiform cells were considered as FLS, which had been proved to excessively proliferate, and the apoptosis was inhibited. To prove the inhibitory effect of berberine on the growth of FLS-RA, MTT and flow apoptosis assays were initially adopted. Our results suggested that berberine inhibited cell proliferation in a time- and dose-dependent manner. Moreover, the berberine doses of 12.5 and 25 *μ*M were adopted for further experiments since the inhibition ratio was close to those of IC20 and IC30 (Figure 2(a)). Besides, berberine promoted the early apoptosis of FLS-RA (Figure 2(b)) and prevented cells from dividing to promote cell cycle arrest at the G0/G1 phase (Figure 2(c)). Besides, RT-PCR results suggested that berberine treatment obviously inhibited the mRNA expression of IL-6 in a dose-dependent manner (Figures 2(d) and 2(e)).

### 3.3. Berberine Altered the Expression Levels of ATX, K-Ras, and c-Raf and the Phosphorylation Levels of ERK1/2 and p38

**Figure 3:**
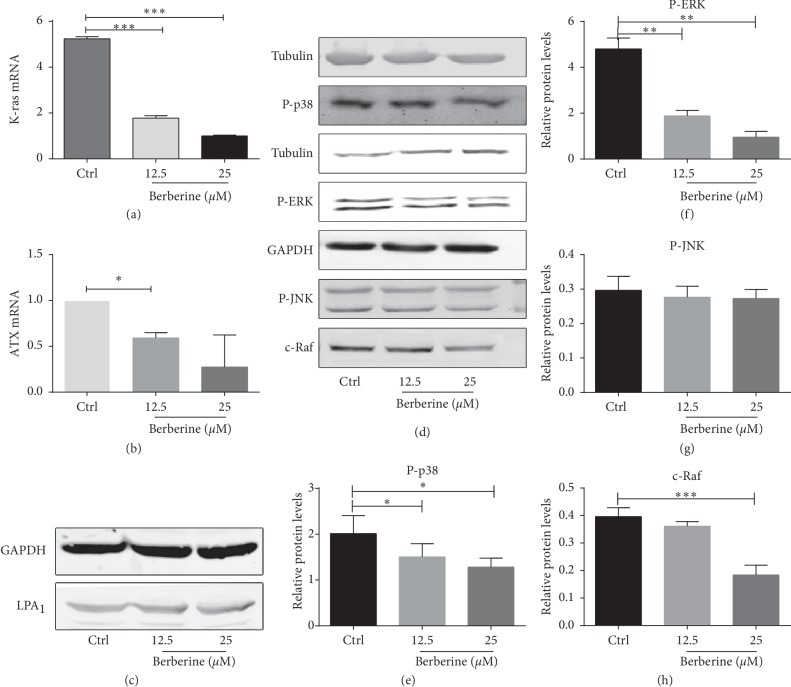
Berberine alters the expression levels of K-ras and c-Raf, as well as the phosphorylation levels of ERK1/2 and p-38 of FLS-RA. (a, b) Berberine treatment at 12.5 and 25 *μ*M for 24 h downregulates the expression of K-ras and ATX, as detected by RT-PCR. (c) Berberine makes no difference to the expression of LPA_1_, as measured by western blotting. (d) The expression levels of c-Raf, P-p38, P-JNK, and P-ERK1/2 are measured through western blotting. Berberine treatment for 24 h downregulates the expression of P-p38 (e), P-ERK1/2 (f), and c-Raf (h) in a dose-dependent manner. (g) The phosphorylation level of JNK shows no significant difference before and after berberine treatment. ^*∗*^*P* < 0.05; ^*∗∗∗*^*P* < 0.001. Ctrl, control group; ATX, autotaxin; and FLS-RA, fibroblast-like synovial cells of rheumatoid arthritis.

To investigate the mechanism by which berberine promoted the apoptosis and inhibited the growth and secretion of proinflammatory factors, the expression levels of K-ras and ATX were measured through RT-PCR, whereas those of LPA_1_, c-Raf, P-p38, P-JNK, and P-ERK1/2 were detected by western blotting, and the aforementioned factors were known to play critical roles in cell growth and proinflammation. Our results suggested that berberine treatment downregulated the expression of K-ras (Figure 3(a)), ATX (Figure 3(b)), c-Raf, P-p38, and P-ERK1/2 in a dose-dependent manner (Figure 3(d)). Interestingly, the LPA_1_ level (Figure 3(c)) and the phosphorylation of JNK remained largely unchanged before and after berberine treatment.

### 3.4. Berberine Potentially Bound to LPA_1_, as Detected by Molecular Docking

**Figure 4:**
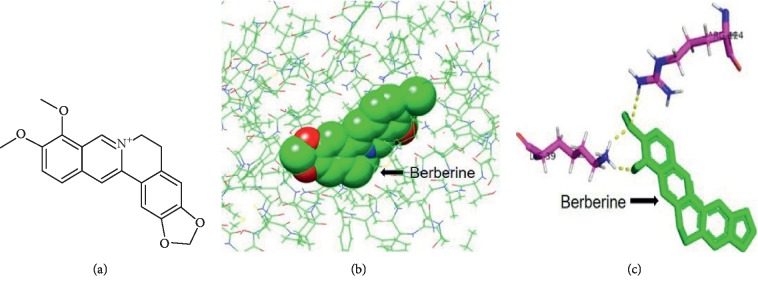
Berberine potentially binds to LPA_1_, as detected by molecular docking. (a) The structure of berberine. (b) The schematic diagram illustrating the combination of berberine with LPA_1_. (c) Berberine potentially binds to Lys39A of LPA_1_ by two hydrogen bonds and to Arg124A by the hydrophobic force. LPA_1,_ lysophosphatidic acid receptor 1; Arg, arginine; and Lys, lysine.

The binding conformation of berberine against LPA_1_ is shown in Figure 4. As can be seen, the lowest binding energy was −6.66. Also, it was found that berberine might bind to Lys39A of LPA_1_ through two hydrogen bonds and to Ala199A by the hydrophobic force, as suggested by molecular docking. This result indicated that berberine might regulate the LPA signaling pathway through LPA_1_.

### 3.5. Berberine Inhibited the Proliferation and Inflammation of FLS-RA through the p38/ERK MAPK Pathway, Partially Mediated by LPA_1_

**Figure 5:**
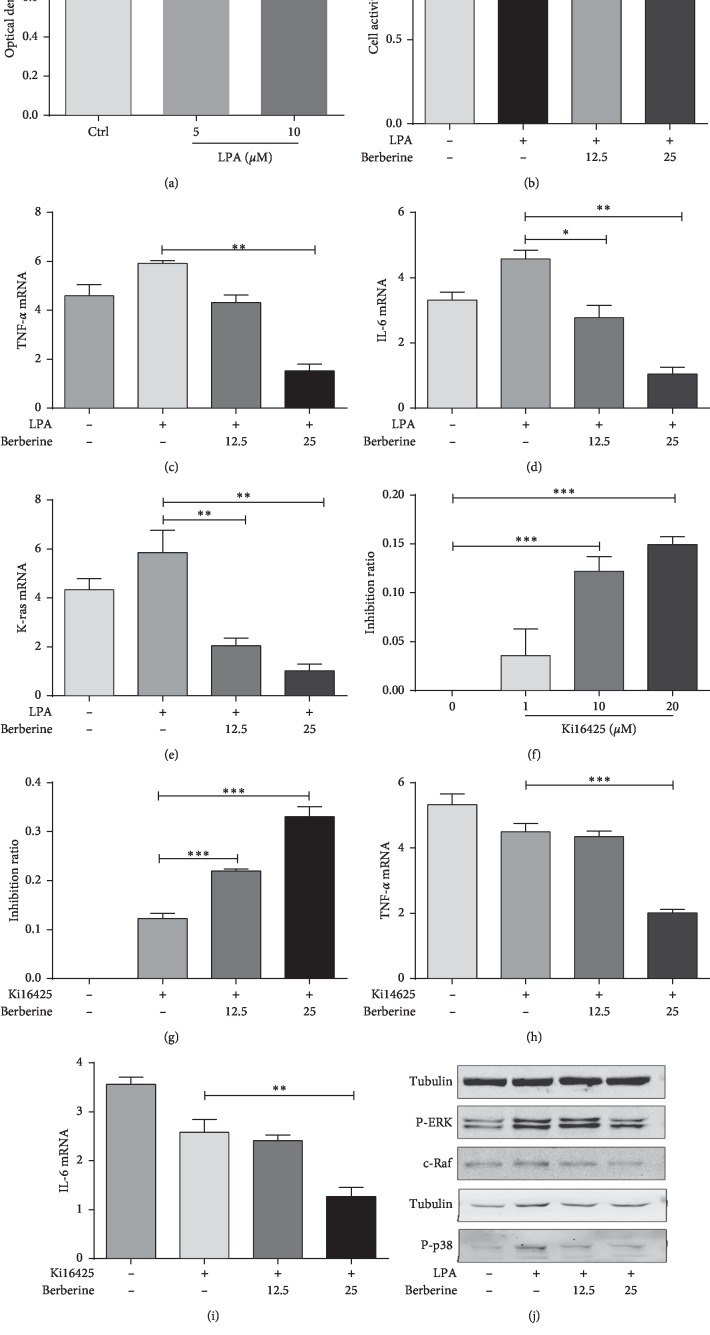
Berberine inhibits the proliferation and inflammation of FLS-RA through the p38/ERK MAPK pathway partially mediated by LPA_1_. (a) LPA treatment for 24 h promotes cell proliferation, as detected by MTT assay. (b) The LPA-induced pro-proliferative effect is reversed by the addition of berberine, as measured by MTT assay. (c, d, e) Pretreatment with berberine remarkably restrains the expression of TNF-*α*, IL-6, and K-ras, and such condition is not obviously changed after the addition of berberine. (f) Ki16425 inhibits cell proliferation, as indicated by MTT assay. (g) 1 h Ki16425 pretreatment in FLS-RA before incubation with berberine for 24 h measured by MTT assay. (h, i) Ki16425 pretreatment in FLS-RA distinctly downregulates the expression of TNF-*α* and IL-6, and such condition is not obviously changed after the addition of berberine. (j) Western blotting and RT-PCR show that LPA pretreatment in FLS-RA significantly promotes the expression of K-ras, c-Raf, P-p38, and P-ERK1/2, while such condition is reversed in the presence of berberine pretreatment. ^*∗*^*P* < 0.05, ^*∗∗*^*P* < 0.01, and ^*∗∗∗*^*P* < 0.001. Ctrl, control group; LPA_1_, lysophosphatidic acid receptor 1; LPA, lysophosphatidic acid; FLS-RA, fibroblast-like synovial cells of rheumatoid arthritis.

To select the appropriate contents of LPA and Ki16425, FLS-RA were incubated with different doses of LPA and Ki16425 for 24 h, respectively. According to our results, 10 *μ*M 13% LPA promoted cell proliferation, as suggested by MTT assay (Figure 5(a)), while such pro-proliferative effect was reversed by the addition of berberine (Figure 5(b)). The effect of Ki16425 was just opposite compared with that of LPA (Figure 5(f)), while pretreatment with Ki16425 or not seemed to make no difference in the presence of berberine treatment (Figure 5(g)). Western blotting and RT-PCR indicated that LPA pretreatment in FLS-RA evidently promoted the expression of K-ras, c-Raf, P-p38, and P-ERK1/2, while pretreatment with berberine reversed the above condition (Figures 5(c), 5(d), and 5(j)). Pretreatment with Ki16425 in FLS-RA significantly downregulated the expression of K-ras, TNF-*α*, and IL-6, and such condition was not markedly changed after the addition of berberine (Figures 5(h) and 5(i)).

## 4. Discussion

Dyslipidemia and CVD appear much earlier in RA patients, which are also observed even before the diagnosis of RA. Retrospective trails suggest that the risk of acute myocardial infarction (MI) in RA after adjusting for CV risk factors increases by up to 1.6 times compared with that in the healthy control [15]. Some epidemiological studies indicate that RA is an independent risk factor of CVD. The CVD mortality decreases following antirheumatism treatment, but it remains relatively high, and CVD events occur earlier in RA patients than in the general population [10]. One possible hypothesis is that antirheumatoid therapy increases the traditional CVD risk factors, such as elevating the LDL and triglyceride levels. It is demonstrated in a meta-analysis that both nonselective NSAIDs and corticosteroids have CVD effects on RA [16]. Despite the remarkable progresses made in RA therapeutic regimen over the past decades, the standardized CV mortality remains unchanged as anticipated, suggesting that it is urgent to target the reduction of RA-CVD mortality [4]. The pathophysiology of CVD in RA involves both chronic inflammation and considerable changes in lipids, among which, the former is considered as a major CV risk factor. Plenty of evidence indicates that some increased proinflammatory molecules, such as high-sensitive C-reactive protein (hs-CRP), TNF-*α*, and IL-6, will aggravate joint destruction and induce other CV risk factors, including changes in lipid levels, metabolic syndrome insulin resistance, and endothelial dysfunction [17–19]. According to a meta-analysis, diabetes mellitus is more prevalent among RA population since chronic lipid inflammation interacts with the inflammation of arthritis [20]. Interestingly, the levels of circulating lipids are reduced in a disease associated with the increased CVD risk, namely, the lipid paradox, which is in contrast to that in the general population [8]. One explanation is that both leukocytes and macrophages engulf the inflammatory lipids and release them after anti-inflammation. However, with the deepening of research, changes in the levels and composition of lipid subfractions may be more related to the inflammation-related CVD compared with the overall lipid level [21]. Although LDL-C declines in RA, small LDL particles are observed at an elevated level in those patients [22]. Besides, the proinflammatory HDL phenotype is related to the elevated molecular levels, suggesting that the anti-inflammation of HLD, rather than the absolute HDL levels, may serve as a more sensitive marker of CVD.

LPA, one of the most prominent glycerophospholipids, has been proven as a novel class of inflammatory lipid and the primary platelet-activating fat among atherosclerotic patients [23]. At present, two pathways have been observed for the cellular formation of LPA. Extracellularly, LPA is mainly formed and released by the activated platelets; in addition, it can also be produced under the action of secretory phospholipase A2 on microvesicles derived from blood cells in the presence of inflammatory stimulation. In the inflammatory response, platelets are activated by invading the pathogens or inflammatory factors, which can thereby produce LPA. Both LPA and the activation of platelets are involved in processes of atherosclerosis, which are closely correlated with CVD. It was confirmed in our study that the LPA levels in RA were obviously higher than those in the healthy control, and there was no statistical difference between the serum and synovial fluid in RA (Figure 1(a)), which has suggested one factor of synovial hyperplasia and high CVD risk in RA apart from the high inflammatory burden. Unfortunately, the synovial fluids of other diseases were not obtained as a control to prove that the RA joint was exposed to the high level LPA microenvironment.

A majority of extracellular LPA originate from the enzymatic action of ATX [24]. ATX, which is distributed in blood and other body fluids, is found in large quantities in the activated arthritic synovial fluid from human patients and the collagen-induced arthritis model [25]. Genetic knock-off of ATX from SFs attenuates the arthritis animal models. TNF, one of the major proinflammatory factors in RA, has been confirmed to induce ATX expression from FLS, which may account for the presence of more LPA in plasma among RA patients [24, 25]. Our findings also indicated that berberine inhibited ATX expression (Figure 3(b)), but it remains unclear about whether berberine reduces the secretion of LPA, which should be further verified in future studies. LPA promotes the proliferation of several cell types, and the LPA signaling has been proven previously to participate in the inflammatory response of FLS-RA [26]. FLS, one of the synovium components, is known as the major effector cell in disease development to release the metal matrix protease and format the inflammatory cascade. Stimulated by the inflammatory factors, FLS acquires the excessive proliferation phenotype and apoptosis resistance. In our study, LPA promoted cell proliferation by 13%. Furthermore, LPA is verified to suppress the apoptosis of the human T-lymphoblast cell line [27]. Notably, the effects of LPA are dependent on G-protein and the MAPK signaling by activating the LPA receptors [26]. Typically, the reported expression types of LPA receptors in human RA SFs are slightly different. For instance, the LPA_1-3_ mRNA is expressed in one study, while in the other one study, only LPA_1_ and LPA_2_ mRNA is detected in FLS-RA [28]. It is found that the LPA_1_-deficient mice will not develop arthritis after immunization with type II collagen (CII), and the LPA_1_ antagonist can also ameliorate murine CIA. The LPA_1_ abrogation is associated with decreases in cell infiltration, joint bone destruction, and interleukin-17 (IL-17) production from the CII-stimulated splenocytes [29]. All studies have reached a consensus that LPA_1_ is an essential receptor in FLS-RA. Meanwhile, the elevated LPA_1_ level in RA was also visible in this study, but berberine shows no effect on lowering its expression (Figure 3(c)).

The biological activities of LPA and the G-protein-coupled receptor agonist mainly take effect by activating the small GTPases Ras, leading to the activation of the MAPK cascade. Typically, the MAPK cascades are involved in the inflammation, proliferation, and joint destruction of RA [30]. ERK activation is localized around synovial vessels, whereas p38 MAPK activation is observed in the synovial lining layer and synovial endothelial cells [31]. FR167653, a potent inhibitor of p38 MAPK, has been identified to prevent arthritis onset in the prophylactic treatment model, while suppressing joint destruction progression in a treatment model [32]. Besides, berberine is found to protect against myocardial ischemia-reperfusion injury through attenuating the NF-kappa B and JNK signaling pathways, suggesting that the MAPK pathway may be one of the mechanisms of berberine in RA [33]. It was observed in this study that berberine suppressed the production of inflammatory mediators and the excessive proliferation of FLS-RA. Additionally, berberine also inhibited the expression of K-ras and c-Raf, as well as the phosphorylation of ERK1/2 and p-38 (Figure 3).

To further understand the molecular mechanism of berberine in regulating LPA function, the binding of berberine to the potent target was simulated through network pharmacology, and it was discovered that berberine might bind to LPA_1_ at the site consistent with the known ligand ONO978030 (Figure 4). According to our study, berberine reversed the LPA-induced changes in phenotypes and the MAPK pathways, which played a similar role to that of Ki16425, revealing that berberine competitively blocked LPA_1_. Interestingly, berberine continued to function when Ki16425 had entirely blocked LPA_1_, prompting that berberine might activate other signaling pathways.

Berberine, a clinically important natural alkaloid, has been recognized for its excellent antidiarrhoea and antidiabetic properties [34]. Nowadays, plenty of studies suggest that berberine exerts other pharmacological effects, such as anti-inflammation and antimigration. Besides, it alleviates the severity of RA rats and increases the levels of anti-inflammatory factors IL-10 and TGF-*β* [32]. Additionally, splenic DCs are more sensitive to berberine than T and B cells, showing that berberine selectively induces the apoptosis of DCs [35]. Moreover, berberine is also suggested in studies to ameliorate the collagen-induced arthritis in rats, which is achieved through suppressing Th17 cell responses via inducing cortistatin in the gut [36]. The aforementioned studies reveal that berberine exerts various pharmacological antirheumatism activities through multiple targets. Otherwise, berberine is also shown to possess a significant lipid-regulating effect. Several clinical trials suggest that berberine improves the fatty liver disease, and it may serve as an effective drug to improve the lipid profile [37, 38]. Apo B is an integral part of the connection between LDL particles and LDL receptor, which has been recognized as a CV risk factor. Moreover, berberine has been shown to reduce the apo B level, thus reducing cholesterol absorption [39]. It is shown in a meta-analysis that the serum TG and total cholesterol (TC) levels are markedly reduced in patients with nonalcoholic fatty liver disease after taking berberine hydrochloride tablets at a dose of 1.5 g/d [40].

Inflammation alters the lipid level, while lipids can in turn aggravate inflammation and the CVD risk. Reducing inflammation can lower the CV risk, which may clarify that RA therapies can decrease the risk of CVD; but on the contrary, RA therapies can also raise the traditional CVD risk factors, such as LDL and TC in remission, which are considered as adverse [41]. LPA acts as a kind of proinflammatory lipid, and attention should be paid to the bioactivity of endothelial dysfunction, hypercoagulability, and early atherosclerosis with unstable plaque formation in RA patients with high CVD risk. Berberine and analogues can be reasonably regarded as the potential drugs to restrain LPA activity and the anti-inflammatory effect of berberine in FLS-RA among RA patients with high CVD risk.

In conclusion, it is identified in this study that LPA has mitogenic and proinflammatory effects on FLS-RA. In addition, berberine modulates the function of active lipid LPA to inhibit the proliferation and inflammation of FLS-RA via blocking the p38/ERK MAPK pathway mediated by LPA_1_, suggesting that berberine has potential lipid-regulating, antiarthritis, and synovial hyperplasia inhibition activities against RA. This study also provides a promising therapeutic target for clinical drug development for those RA patients with dyslipidemia and high CVD risk.

## Data Availability

The data used to support the findings of this study are available from the corresponding author upon request.
